# A Tribute to the Medical Profession

**Published:** 1908-07

**Authors:** Victoria A. H. Duggan

**Affiliations:** San Antonio, Texas


For Texas Medical Journal.

### A Tribute to the Medical Profession.

### THE OLD PHYSICIAN.

Once on a time, in the long ago,
There lived a physician with hair like snow.
He had made his "rounds" upon each day,
But now, on his dying bed he lay,
Breathing, sighing,
Slowly dying
This physician worn and grey.

As the war-horse after battle
Thinks he hears the din and rattle,
Bugle calls, tho' he is dying,
Makes an effort, then falls, sighing,
Trying, sighing,
Dying, trying,
Still he's done the best he can.

Hark! He murmurs in unrest.
His faithful wife, with tears suppressed
Tries to catch what he is saying,
Meanwhile she is softly praying,
Weeping, praying,
Praying, weeping
As she catches what he says.

"Wife, they're calling; help me dress.
Some poor soul's in dire distress.
Get my hat and cane for me.
Help me go. Ah, don't you see,—
They are calling.
Hurry, darling!"
Then he sinks back, silently.

As he sank back on his pillows
Breathing now, like ocean billows

This old man of three score ten
Answered to the great Amen.
Then silence fell,
And all was well
With one of God's own noblemen.

Who are conquerors in this life
Who are victors in the strife?
'Tis these men, who live for others,—
Who count all men, as their brothers,
Giving, living,
Dying, giving
They have won the crown of life!

VICTORIA A. H. DUGGAN.

San Antonio, Texas, June 30, 1908.
[In memory of her father.—ED.]

---

### Texas State Medical Association.

President Cummings announces the following committees and section appointments:

*Legislative Committee*—Dr. H. W. Cummings, President, Hearne, ex-officio; Dr. I. C. Chase, Secretary, Fort Worth, ex-officio; Dr. W. B. Russ, San Antonio; Dr. J. A. Holloway, Round Rock; Dr. J. A. Hill, Groveton.

*Memorial Committee*—Dr. C. P. Yeager, Corpus Christi; Dr. John T. Moore, Galveston; Dr. R. E. B. Bledsoe, Somerville.

*Representative, Council Medical Education*—Dr. John T. Moore, Galveston.

*Section on State Medicine and Public Hygiene*—Dr. F. E. Daniel, Chairman, Austin; Dr. L. B. Bibb, Secretary, Austin.

*Section on Surgery*—Dr. F. L. Barnes, Chairman, Trinity; Dr. W. G. Jameson, Secretary, Palestine.

*Section on Medicine and Diseases of Children*—Dr. J. H. Wysong, Chairman, Hico; Dr. S. P. Rice, Secretary, Marlin.

*Section on Gynecology and Obstetrics*—Dr. J. W. Hale, Chairman, Waco; Dr. H. M. Doolittle, Secretary, Dallas.

*Section on Psychology and Medical Jurisprudence*—Dr. G. H. Moody, Chairman, San Antonio; Dr. F. U. Painter, Secretary, Pilot Point.

*Section on Pathology*—Dr. J. M. Frazier, Chairman, Belton; Dr. K. H. Beall, Secretary, Fort Worth.

*Section on Otology and Ophthalmology*—Dr. E. H. Cary, Chairman, Dallas; Dr. Joseph Mullen, Secretary, Houston.

